# Fish *Iridoviridae*: infection, vaccination and immune response

**DOI:** 10.1186/s13567-024-01347-1

**Published:** 2024-07-15

**Authors:** Rocío Leiva-Rebollo, Alejandro M. Labella, Juan Gémez-Mata, Dolores Castro, Juan J. Borrego

**Affiliations:** 1 Departamento de Microbiología, Universidad de Málaga, Málaga, Spain; 2 Department of Microbiology, Icahn School of Medicine at Mount Sinai, New York, NY USA; 3 Global Health and Emerging Pathogens Institute, Icahn School of Medicine at Mount Sinai, New York, NY USA

**Keywords:** Fish *Iridoviridae*, immune response, aquaculture, vaccines, viral infection

## Abstract

Each year, due to climate change, an increasing number of new pathogens are being discovered and studied, leading to an increase in the number of known diseases affecting various fish species in different regions of the world. Viruses from the family *Iridoviridae*, which consist of the genera *Megalocytivirus*, *Lymphocystivirus*, and *Ranavirus*, cause epizootic outbreaks in farmed and wild, marine, and freshwater fish species (including ornamental fish). Diseases caused by fish viruses of the family *Iridoviridae* have a significant economic impact, especially in the aquaculture sector. Consequently, vaccines have been developed in recent decades, and their administration methods have improved. To date, various types of vaccines are available to control and prevent *Iridoviridae* infections in fish populations. Notably, two vaccines, specifically targeting Red Sea bream iridoviral disease and iridoviruses (formalin-killed vaccine and AQUAVAC^®^ IridoV, respectively), are commercially available. In addition to exploring these themes, this review examines the immune responses in fish following viral infections or vaccination procedures. In general, the evasion mechanisms observed in iridovirus infections are characterised by a systemic absence of inflammatory responses and a reduction in the expression of genes associated with the adaptive immune response. Finally, this review also explores prophylactic procedure trends in fish vaccination strategies, focusing on future advances in the field.

**Supplementary Information:**

The online version contains supplementary material available at 10.1186/s13567-024-01347-1.

## Introduction

Among the many viral diseases that threaten the economic stability of the aquaculture sector, iridoviruses are emerging pathogens with a pervasive presence across diverse environmental niches and host species. Significant mortality rates characterise these diseases, affecting both wild and farmed animals and establishing viral reservoirs amongst those populations [1]. To mitigate this potentially unsafe impact, it is necessary to implement biosecurity measures that focus on four main areas: fish, pathogens, environment, and personnel management. These management factors involve selecting pathogen-free broodstock, feeding optimisation, rigorous hygiene and sanitation protocols for facilities, the use of approved, available drugs, methods for microbial pathogens detection, and the disinfection of fertilised eggs, rearing water, and effluents to ensure healthy stocks for fish farming [2]. Furthermore, it is essential to improve the resilience and health of aquaculture systems against iridovirus infections by integrating prophylactic measures, notably through developing and administering fish vaccines [3]. Creating effective and state-of-the-art vaccines requires careful consideration of various factors, including identifying suitable vaccine antigens, the vaccine type, and the administration protocols. Crucially, a profound understanding of the fish immune response is essential for guiding the selection of immune response pathways and the essential determinant genes associated with virus inhibition or elimination. Consequently, studies such as transcriptomic analyses are gaining increasing traction as they offer valuable insights into the intricacies of immune responses across diverse fish species, thereby facilitating the identification of potential therapeutic targets for vaccine development [4].

Numerous studies carried out over recent decades have concluded that fish vaccination is an effective, easy, and inexpensive method of controlling microbial pathologies and preventing many re-emerging diseases in aquaculture systems. Aquaculture most crucial environmentally friendly disease control strategy is the fish vaccination procedure. It helps prevent and control viral diseases and significantly reduces reliance on antibiotics. Moreover, immunostimulants can enhance the effect of vaccines by boosting the immune mechanisms in fish and providing specific long-term protection against one particular microbial infection based on enhancing innate and adaptive immune responses [5].

Building upon these considerations, the main goal of this review is to comprehensively analyse the existing vaccines developed against iridoviral diseases in fish within the aquaculture sector. It will address the challenges posed by both emerging and re-emerging diseases in aquaculture. Additionally, it aims to consolidate the available information regarding fish immune responses to iridovirus infections, their subsequent immune responses after vaccination, and the way forward for vaccine development.

## Type of fish virus vaccines and their delivery methods

A vaccine is a biologically based preparation designed to enhance immunity against a specific infectious agent by triggering an innate and/or adaptive immune response to a particular antigen derived from or present in the disease-causing pathogen. Presently, 26 licensed fish vaccines are commercially available worldwide, catering to various fish species and offering protection against a range of fish pathogens, including certain families of viruses such as rhabdoviruses, birnaviruses, orthomyxoviruses, alphaviruses, alloherpesviruses, and iridoviruses [6]. Vaccines are typically administered through various routes, including oral, injection (intraperitoneal or intramuscular), and immersion [7]. Selecting the most effective route depends on several factors, such as the pathogen, its mode of infection, the immunological memory status, vaccine production methods, labour costs, and the life phase of the fish. The choice of delivery method may impact the immune response and the level of protection against the target pathogen, as the kinetics and magnitude of the innate immune response vary depending on the vaccination route [8, 9].

Oral vaccination incorporates the fish vaccine into the feed through coating, spraying, or bioencapsulation [10]. Plant and LaPatra reported that this method offers advantages such as cost efficiency (particularly for larger fish), simplicity, and safe administration across different fish sizes and developmental stages while also inducing minimal stress [11]. Additionally, oral vaccination is a convenient means for boosting immunity during grow-out periods in cages or ponds. However, it tends to have lower efficacy due to the limited time and level of defence against antigen destruction, degradation, and absorption by the fish´s gastrointestinal tract. Orally administered vaccines, particularly those containing inactivated whole antigens and a booster vaccine for primary vaccination, elevate the protection against pathogens [12].

Injection methods, specifically intraperitoneal (IP) or intramuscular (IM) routes, offer advantages over immersion vaccination. They require only a small amount of antigen and provide longer-lasting protection to fish. IP injection is considered the most efficient and productive way to immunise fish, often using adjuvants for enhanced protection compared to immersion methods. Recent vaccines have been administered using this route. Conversely, fish farmers prefer IM delivery for DNA vaccination due to its longer protection duration. However, one drawback of injection methods is a higher incidence of stress-induced fish mortality following vaccination [9].

Immersion vaccination is effective for immunising fish against microbial infection, especially with live, attenuated, or vector formulations. This technique can be carried out through either a dip (a brief immersion for 30 s with a high vaccine concentration) or a bath (several hours with a lower vaccine concentration) [13]. Immersion is widely utilised and recommended for smaller fish due to its effectiveness, rapidity, convenience, low stress-inducing levels, and its cost-effectiveness. However, one disadvantage is the short duration of fish immunity, ranging from 3 to 12 months, often necessitating booster doses. Moreover, immersion vaccination is impractical for large fish due to longer processing times, increased costs, elevated stress levels, and difficulties administering multiple immune stimulants and adjuvants [14].

Inactivated (or ‘killed’ vaccines) are derived from microbial suspensions treated with physical or chemical agents (e.g., heat, UV, formaldehyde, ethylamine, or β-propiolactone). These agents block microbial nucleic acid replication while preserving antigenicity. Despite being easy to prepare, exhibiting high stability of immunogenicity in field conditions, and being relatively inexpensive, these vaccines require high inoculation doses and may induce toxic reactions due to immune-enhancing adjuvants. Additionally, the denaturation of proteins can reduce immunogenicity, leading to weaker or shorter-lived immunity [14, 15]. To address this, adjuvants or multiple booster immunisations may be necessary.

Attenuated vaccines consist of live microorganisms lacking the ability to cause a productive infection. They tend to be more immunogenic than inactivated vaccines due to their ability to proliferate, provide cellular and humoral immunity, and ease of host entry. However, they have a short validity period, poor thermal stability, and risk reverting to pathogenic forms and establishing infection in immunocompromised individuals [9]. Various methods, including natural selection, serial passages in cell line cultures, gene deletion, and reverse genetics, are used to obtain avirulent virus strains for attenuated vaccines. Currently, only one commercial vaccine by KoVax is available in Israel based on a live attenuated virus. It targets the carp disease koi herpesvirus (KHV) and is administered through intraperitoneal, oral, or immersion routes [16].

Subunit vaccines utilise only immunogenic components of pathogens, and there is no risk of pathogenicity to hosts or non-target species as they cannot replicate within the host. These components can be isolated directly from the pathogen or produced using recombinant expression vectors such as *Escherichia coli*, yeast, insect cells, and cabbage worms, or designed using in silico analysis. As these vaccines may elicit a weaker immune response, they often require adjuvants or multiple booster immunisations for long-term immunity [17].

DNA vaccines, consisting of plasmid carrying specific antigenic genes, are safe and advantageous, requiring only immunogenic parts of the pathogen. Moreover, they have other advantages such as the potential to co-administer multivalent vaccines, production flexibility that is scalable and cost competitive, and stability in storage due to an elevation of chemical stability of the plasmid DNA [18, 19]. DNA vaccines do not require adjuvants and can effectively activate both cellular and humoral immunity. DNA vaccines have been developed against various fish viruses, including rhabdoviruses, herpesviruses, orthomyxoviruses, betanodavirus, togaviruses, birnaviruses, and iridovirus. A recombinant DNA vaccine containing the puK-SPDV-poly2#1 plasmid, encoding several proteins from SAV-3 (CLYNAV), has been approved in Norway and the EU to combat pancreatic disease [20]. Remarkably, research indicates that the method of vaccine administration significantly impacts the immune response to DNA vaccines, with oral and immersion routes demonstrating superior fish immunological protection [21, 22].

Newer RNA-based vaccines offer several advantages over traditional fish vaccines. They have enhanced immunogenicity, easier degradation by normal cellular processes, and improved safety due to the absence of infection or insertional mutagenesis risks [23]. Currently, two major types of RNA-based vaccines exist; these are conventional, non-replicating mRNA and self-amplifying mRNA (i.e., replicons). Self-amplifying RNA vaccines, by replacing genes for virus structural proteins with fish pathogen antigens, show promise in protecting against various fish viral diseases [24].

Mucosal vaccines are gaining attention in aquaculture for their longer immunity periods. They may elicit protective responses at mucosal surfaces, inhibiting pathogen replication at the initial site. Live vector vaccines, a subgroup, use genetically modified non-pathogenic viruses as carriers to express immune-related antigens, including intestinal mucosal immune responses. A significant benefit of live vector vaccines is their ability to effectively stimulate cellular, humoral, and mucosal immunisation by inducing antigen expression in vivo [25].

Nanoparticles are increasingly being considered as potential vaccine candidates for use in aquaculture. Their small size allows for distribution throughout the organism via the circulatory system, entering target cells such as capillaries. Nanovaccines offer additional advantages beyond enhanced immune system activation without booster doses. They do not require cold chain maintenance, simplifying storage and distribution logistics and reducing the costs and logistical challenges associated with traditional vaccines [26].

## *Iridoviridae*: taxonomy and host range

The family *Iridoviridae* are composed of large double-stranded DNA (dsDNA) viruses with icosahedral symmetry and diameters ranging from 120 to 200 nm. Members of this family have a broad host range and infect vertebrates, including bony fish, amphibians, and reptiles [27] (Table 1). The genera can be distinguished by the following characteristics: guanine-cytosine (GC) content, nucleotide and protein sequence identity, phylogenetic relatedness, disease manifestations, and antigenicity [27–30].

**Table 1:** **Taxonomy of the family ***Iridoviridae*** and its features**

Family	Subfamily	Genus	Species	Genotypes	Clades	Host	Optimal replication T^º^	Mortality	References
*Iridoviridae*	*Alphairidovirinae*	*Megalocytivirus*	ISKNV SDDV	RSIV TRBIV ISKNV	ISKNV-I/-II TRBIV-I/-II RSIV-I/-II	Fish	20–25 °C	20–60%	[33–36]
		*Lymphocystivirus*	LCDV-2/LCDV-C LCDV-1 LCDV-3/ LCDV-Sa LCDV-4	I- LCDV-1 II- LCDV-C III- LCDV-RF IV- LCDV-RC and LCDV-SB V-LCDV-CB VI for gourami VII- LCDV-Sa and LCDV-SSE VIII- largemouth bass IX- American yellow perch X- LCDV-WC	–	Fish	20 °C	Not mortality	[124, 125]
		*Ranavirus*	FV3 ATV CMTV EHNV ENARV SCRV SGIV	–	–	Fish Amphibian Reptile	12–32 °C	0–100%	[126, 127]

ISKNV: *Infectious spleen and kidney necrosis virus*; SDDV: *Scale drop disease virus*; LCDV: *Lymphocystis disease virus*; FV3: *Frog virus* 3; ATV*: Ambystoma tigrinum virus*; CMTV: *Common midwife toad virus*, including *common midwife toad virus NL*, *common midwife toad virus E*, *andrias davidianus ranavirus*, *testudo hermanni ranavirus*, *pike perch iridovirus*, *pelophylax esculentus virus*, and *rana esculenta virus*; EHNV: *Epizootic haematopoietic necrosis virus*, including *epizootic haematopoietic necrosis virus*, *European catfish virus*, or *European sheatfish* (ESV); ENARV: *European North Atlantic ranavirus*; SCRV: *Santee Cooper ranavirus* including the *largemouth bass virus*, *guppy virus 6*, and *doctor fish virus* (DFV); SGIV: *Singapore grouper iridovirus* including *Singapore grouper iridovirus* and *grouper iridovirus* (GIV); RSIV: *Red sea bream iridovirus*; TRBIV: *Turbot reddish body iridovirus*; genotype I: LCDV-1 *European flounder* isolates; genotype II: LCDV-C *Japanese flounder* isolates; genotype III: LCDV-RF black rockfish isolates; genotype IV: LCDV-RC and LCDV-SB consist of cobia and Japanese sea bass isolates; genotype V: LCDV-CB painted glassfish isolates; VI: Gourami isolates; VII: LCDV-Sa and LCDV-SEE includes gilthead sea bream and Senegalese sole isolates; genotype VIII: largemouth bass isolates; genotype IX includes American yellow perch isolates; and genotype X: LCDV-WC Whitemouth croaker isolates. – there are no genotypes or clades specified. T^º^ This is referred to temperature

### Megalocytiviruses

The genus *Megalocytivirus* comprises two species: *Infectious spleen and kidney necrosis virus* (ISKNV) and *Scale drop disease virus* (SDDV). Furthermore, ISKNV species include a large cluster represented by three genotypes, subdivided into clades I and II, generating six clades (Table 1) [30, 31]. The second cluster includes strains belonging to the SDDV species. An SDDV-close European chub iridovirus (ECIV) and an unclassified three-spined stickleback (TSIV) were recently isolated and classified. They have been proposed as species within the MCVs genus [11, 30, 32]. Megalocytiviruses provoke systemic, often life-threatening diseases, characterised by enlarged cells called ‘inclusion body-bearing cells’ in infected organs and tissues such as the spleen, kidney, gastrointestinal tract, and gills. Inclusion body-bearing cells are hypertrophied cells with large basophilic and granular inclusions that distend the cytoplasm and displace the nucleus. Infected fish are lethargic and show abnormal body colouring, gill petechiae, and histological changes in the spleen, gills, and gut tract. This can all lead to mortality amongst wild and farmed marine and freshwater fish, including ornamental species [33]. This genus is gaining attention because it causes significant mortality that occasionally can reach up to 100% during epizootics in captive fish populations and experimental infections [29, 32, 34, 35] (Table 1). Additional file 1 lists the main species of fish, both marine and freshwater, which have been described as susceptible to infection with megalocytiviruses. Megalocytiviruses are listed by the World Organization for Animal Health (WOAH, formerly the OIE) in chapter 1.3 as the causative agent of red sea bream iridovirus diseases [28, 36–38].

### Lymphocystiviruses

The genus *Lymphocystivirus* contains four species: *Lymphocystis disease virus 1* (LCDV1), *Lymphocystis disease virus 2* (LCDV2, LCDV-C), *Lymphocystis disease virus 3* (LCDV3, LCDV-Sa) and *Lymphocystis disease virus 4* (LCDV-4) (Table 1). LCDV infects more than 100 species of marine and freshwater fish (Additional file 1) [30–41], resulting in wart-like lesions, usually on the external surface of infected fish. These lesions consist of clusters of individual infected cells (about 1 mm in size), mainly on the skin but sometimes in internal organs. Viral morbidity can be high, and, in some cases, large numbers of these lesions can impair the mobility and feeding of infected fish, indirectly contributing to mortality [42, 43]. The duration of virus proliferation in fish is temperature-dependent and highly variable, ranging from five days to nine months.

### Ranaviruses

The genus *Ranavirus* comprises versatile pathogens capable of causing systemic infections in lower vertebrates (classes Reptilia, Amphibia, and Osteichthyes). The broad host range of this genus is a major reason why the WOAH lists ranaviruses as notifiable amphibian and fish pathogens. The *Ranavirus* species can be distinguished by several criteria, including nucleotide sequence identity, phylogeny, host range, and protein and genomic characteristics [29]. According to the International Committee on Taxonomy of Viruses (ICTV) report, this genus comprises seven species (Table 1). It has been hypothesized that ranaviruses have jumped from fish to amphibians and reptiles, the latter probably because they often share habitat with susceptible fish and amphibian species [44]. Brenes et al. demonstrated that ranaviruses can be transmitted through water in cold-blooded vertebrates by cannibalism, parenteral injection, direct contact, or environmental exposure [45]. Outbreaks in some species, such as European perch (*Perca fluviatilis*) can be more lethal than in other species depending on the species, virus and age and health status of the host (Table 1). *Ranavirus* infections cause high levels of morbidity and mortality in their susceptible farmed and wild host species because the mode of transmission promotes a rapid spread of the virus [46]. Typical symptoms of infection in fish include loss of buoyancy, erratic swimming, anorexia, red swollen gills, haemorrhages (internal organs, skin, and eyes), and focal and/or generalized necrosis of the hematopoietic tissues (the spleen, renal hematopoietic tissue, and the liver) and other cells and organs (the gill and gastrointestinal epithelial cells, and the pancreas) [47].

Additional file 1 shows the main host fish species of ranaviruses [29, 48–51].

Table 1 shows the optimal replication in vivo and in vitro and the percentage of mortality associated with these genera.

## Immune responses of infected and vaccinated fish to *Iridoviridae*

### *Iridoviridae* infection and immune response in fish

In line with numerous pathogens, iridoviruses overcome macrophage and host antiviral barriers by down-regulating reactive oxygen species, turning these immune cells into vehicles for virus spread and persistence [52]. Furthermore, another characteristic of various members within the family *Iridoviridae* is the ineffectiveness of several fish IFN/Mx antiviral proteins in preventing infection. For example, the Mx of Japanese flounder (*Paralichthys olivaceus*) can inhibit the replication of two rhabdoviruses (HIRRV and VHSV) but is unable to inhibit the replication of Red Sea bream iridoviral virus (RSIV) [53]. Similarly, Mx proteins from barramundi (*Lates calcarifer*), Senegalese sole (*Solea senegalensis*), and rainbow trout (*Oncorhynchus mykiss*) show no antiviral activity against the megalocytivirus TGIV and the ranaviruses ECV and EHNV, respectively [54–56]. Interestingly, gilthead seabream possesses at least three Mx isoforms that effectively inhibit the replication of the rhabdovirus VHSV and the lymphocystivirus LCDV-Sa (isoform Mx1), both iridoviruses ECV and LCDV-Sa (isoform Mx2) and the rhabdovirus VHSV (isoform Mx3) [57, 58]. This is the first example of a teleost Mx molecule effectively inhibiting DNA virus infection. Therefore, the efficacy of the teleost IFN/Mx response may determine the specific susceptibility of each fish species to viruses. In this manner, after infection with the megalocytivirus TGIV (genotype II, clade 2), high levels of phagocytic basophilic and eosinophilic mononuclear leukocytes have been found to contain TGIV genomic DNA in their nuclei. Certainly, TGIV has evolved complex and regulated strategies to overcome and exploit the host immune cells, constituting a viral immune evasion and dissemination mechanism that is a hallmark of all vertebrate iridoviruses. Iridoviruses also encode genes that interfere with the host immune response. Sequence analysis has identified several putative genes that may help to suppress host immunity. These putative immune evasion proteins include the viral homolog of eIF-2a (vIF-2a), a viral homolog of RNAse III, a virus-encoded CARD (caspase activation and recruitment domain)-containing protein, a viral homolog of steroid dehydrogenase (β-HSD), encode viral proteins (VP48, VP122, VP312) that regulate by inhibiting autophagy, a putative Bcl-2-like protein, and one or more tumour necrosis factor (TNF) receptor homologs [59–61].

Holopainen et al. found that infecting the *Epithelioma papulosum cyprini* (EPC) cell line with the ranaviruses FV3, ECV, DFV, and EHNV, led to different inflammatory gene expression profiles [62]. Specifically, infection with EHNV and FV3 induced the expression of the pro-inflammatory genes *tnfα* and *il1ß*, whereas ECV and DFV induced the transient up-regulation of the immunosuppressive gene transforming growth factor-beta (*tgfß*). Additionally, all ranaviruses induced the expression of apoptotic components and ß2-microglobulin genes, which are critical for surface MHC class I expression and, therefore, for cytotoxic T-cell function. This suggests that the adaptive immune response may also be triggered by these viral infections.

MicroRNAs (miRNAs) are a type of non-coding RNA that play a crucial role in various cellular functions [63]. However, their role in viral infection mechanisms and cellular immune response in fish remains poorly understood [64]. Li et al*.* investigated the role of miR-124 in SGIV-infected, orange-spotted grouper (*Epinephelus coioides*) and the subsequent host immune responses [65]. The expression level of grouper miR-124 was significantly up-regulated after SGIV infection; and although miR-124 does not affect the virus entry, the up-regulated miR-124 could affect the SGIV-induced cytopathic effects (CPEs) and viral gene expressions. Overall, grouper miR-124 could promote viral replication and down-regulate fish immune response by targeting JNK3 (Jun N-terminal kinase) and p38a mitogen-activated protein kinase (MAPK). Similarly, Wu et al. identified numerous megalocytivirus-induced, non-coding RNAs and their interactive targets, highlighting the profound involvement of non-coding RNAs in megalocytivirus infection and host immune responses [66]. It is worth noting that infection suppresses the expression of mir-144, a factor crucial in activating RLR and IRF7 expression, thereby facilitating viral clearance. Additionally, mir-144 indirectly regulates the JACK-STAT pathway and IFNγ, contributing to the immune evasion mechanism. Another significant observation is the activation of mir-409, which inhibits the expression of STAT1, thereby suppressing the inflammatory response and promoting viral infection.

Several microarray studies have investigated the transcriptional response of fish to iridovirus infection. Thus, Huang et al. investigated differentially expressed genes (DEGs) in the spleen of the orange-spotted grouper) infected with the ranavirus SGIV [67]. Further KEGG (Kyoto Encyclopedia Genes and Genomes) analysis revealed that the cellular metabolism, and intracellular immune signaling pathways, were present in the infected libraries. Certain genes associated with the MAPK, chemokine, toll-like receptor, and RIG-I (retinoic acid-inducible gene I) signaling pathways were altered in response to SGIV infection.

Park et al*.* studied the DEGs in the spleen of rock bream (*Oplegnathus fasciatus*) infected with the megalocytivirus RBIV (genotype II, clade 2) [68]. Sequencing of the whole transcriptome in infected fish showed that the DEGs were immune-related genes such as interferon-induced and Fc receptor-like. Interleukin-10, perforin-1, and inhibitor of nuclear factor κ-β kinase, complement system (except C4) genes were up-regulated; but IL1β, λ-chain of immunoglobulin (Ig), α-chain Ig, and complement factor H were down-regulated.

Carballo et al. experimentally infected Senegalese sole with the lymphocystivirus LCDV-Sa and studied a set of DEGs in the kidney and intestine for 15 days [69]. They found that LCDV-Sa infection activated immune-related genes, such as interferons, cytokines and their receptors, chemokines, prostaglandins, lysozyme, hepcidin, complement fractions, and several clusters of differentiation of the antigens (*cd4*, *cd8a*, and *cd8b*). These results are a systemic host defence response to viral infection. Previously, Hu et al. also found that several IFN-related genes (*irf3*, *irf5*, *irf7*, *irf8,* and *irf9*) were induced in several organs of Japanese flounder after poly(I:C) or LCDV-C challenge [70].

Liu et al. compared the immune responses of groupers to infection with the megalocytivirus TGIV and the ranavirus GIV [71]. A total of 17 common and five specific pathways were found to be significantly differentially expressed after infection with both iridoviruses. TGIV infection activated the spliceosomal pathway, whereas the glycolysis/gluconeogenesis pathway was associated with TGIV infection, which may explain the different pathologies and symptoms induced by these viruses.

Leiva-Rebollo et al. analysed the immune gene expression response in gilthead seabream (*Sparus aurata*) that were infected with the lymphocystivirus LCDV-Sa [72]. They found viral DNA and transcripts in all the inoculated fish, demonstrating that the virus is capable of causing systemic and asymptomatic infections. The expression of 23 immune-related genes was analysed in the head kidney and intestine. Of these, five IFN-I-related genes (*ifn*, *irf3*, *mx2*, *mx3*, and *isg15*), and two of the interleukin genes (*il10* and *ck10*) were up-regulated, while genes related to the inflammatory process (*tnfα, il1ß*, *il6*, *casp1*) were neither differentially expressed nor down-regulated in the head kidney. The expression profile in the intestine differed in relation to type I INF-related genes. An up-regulated gene for complement *C3* and immunoglobulin heavy constant mu (*ighm*) expression was detected in both organs. Finally, the transcription of *nccrp1* and *mhcIIα* was induced in the head kidney, whereas *tcrb* expression was down-regulated in both organs. In short, LCDV-Sa triggers an immune response in this particular fish species. This response is characterised by an activation of the type I IFN system and a lack of systemic inflammatory response. This may be connected to the virus’s observed persistence in that fish species.

In another study, Carballo et al. infected Senegalese sole post-larvae with the lymphocystivirus LCDV-Sa using two routes: artemia as a vehicle of feed and immersion [73]. An expression analysis of 22 genes related to the innate immune defence system showed apparent differences depending on both the route of infection and the time course of the response. Most antiviral defence genes (proinflammatory cytokines, complement, lysozyme, and T-cell markers) were rapidly induced in the feed-infected post-larvae. The most defensive genes were induced later in the post-larvae that were infected through immersion, in contrast. These results confirmed horizontal waterborne and feed transmission of LCDV-Sa, although with different patterns of histopathological damages, virus distribution, and route-specific expression gene profiles.

The transcriptional responses of orange-spotted grouper to the ranavirus SGIV infection were studied by Yang et al. [74]. KEGG analysis showed that two immune-related pathways were overexpressed: the p53 (related to cell cycle, cellular senescence, and apoptosis) and the peroxisome proliferator-activated receptor (PPAR). The latter is a regulator of innate and adaptive immunity that stimulates an anti-inflammatory activity in several cell types by inhibiting the expression of *AP-1* and *NF-*κ*B* inflammatory genes. Furthermore, a comparable phenomenon was observed in grouper infected with SGIV, where the up-regulation of Krϋppel-like factor 9 (KLF9) and proprotein convertase subtilisin/kexin type 9 (PCSK9) was noted. This up-regulation inhibited the expression of numerous interferon-related cytokines and inflammatory cytokines, thereby facilitating the expression of SGIV-related genes (*mcp*, *litat*, *icp-18*, and *vp19*) [75, 76]. Therefore, susceptibility or resistance to fish diseases caused by iridoviruses may be linked to various pathways, gene expressions, and genetic markers.

The study by Kim et al. investigated the transcriptomic changes induced by the megalocytivirus RBIV infection in the spleen rock bream fish [77]. The DEGs associated with viral infection included unigenes of the cell cycle, DNA replication, transcription, translation, glycolysis activity, and endogenous apoptosis. Several unigenes exhibited a significant decrease in expression, such as the lymphocyte-mediated immune system, antigen presentation, and platelet activation. These results enhance RBIV infection and compromise host defence. The authors also found an overexpressed gene in the infective course, the gene *hub* (a pre-mRNA processing factor 19), which could be a potential candidate for fish vaccination studies against these viruses.

Recently, Domingos et al. investigated the expression of immune genes in barramundi following infection with the megalocytivirus SDDV [78]. The predominantly activated genes found were those related to innate immunity, such as pattern recognition (lectin, chemokine, and interleukin receptors), inflammatory cytokines, TNF, chemokines, complement factors, and immune signal transduction adaptor CD molecules. In contrast, gene families associated with the adaptive immune response (B- and T-cell receptors and MHC) were significantly down-regulated in infected barramundi. According to these results, megalocytivirus infection induces the activation of genes related to innate immunity but down-regulates genes associated with adaptive immunity.

Zheng et al. studied the giant grouper’s (*Epinephelus lanceolatus*) antiviral immune response to the spotted knifejaw iridovirus SKIV (genotype II, clade 2) [79]. KEGG analysis showed that several innate immune and signalling pathways were significantly activated in response to SKIV infection, potentially synergistically contributing to viral clearance. Furthermore, the authors suggest that IRF3 and IRF7 may be involved in the host fish ability to resist viral infection. A recent study conducted by Niu et al*.* in mandarin fish infected by ISKNV revealed that the expression of TRIM59 plays a pivotal role in determining the function of IRF3 and IRF7, exhibiting antiviral activity. Generally, TRIM59 negatively regulates ISKN infection and the expression of IRF3/IRF7-mediated signal genes [80]. In addition, a correlation between the methylated status of the genome of ISKNV and its ability to evade the immune system has recently been discovered, suggesting that an increase in methylation levels allows the virus to sidestep host immune responses [81].

Guo et al*.* performed a transcriptomic analysis to determine the molecular mechanisms induced by ranavirus SGIV infection in grouper spleen cells [82]. SGIV infection activated more than 100 DEGs, the most important of which were the cytoskeleton signalling pathway (involved in the cell rounding during CPE in infected cells) and the MAPK signalling pathway (related to SGIV-induced cell death). In addition, during viral infection, a MAPK gene involved in virus assembly and replication (*c-Jun*) was expressed. On the contrary, most DEGs involved in the immune response (IFNs and inflammatory signalling) were down-regulated during SGIV infection, which may indicate the virus's potential immune evasion mechanism.

### Immune response of fish to *Iridoviridae* vaccination

Table 2 details the different types of vaccines developed to prevent or reduce infections caused by viruses in the subfamily *Alphairidovirinae*. There are currently only a few commercially available vaccines, such as the formalin-killed vaccine for RSIVD in Japan and AQUAVAC^®^ IridoV in Singapore. The AQUAVAC^®^ IridoV vaccine has been shown to provide immunity against iridoviruses [83, 84].

**Table 2:** **Global aquaculture fish vaccines development in the subfamily** ***Alphairidovirinae***

Pathogen	Fish species		Vaccine type	Antigens	Delivery	Country	Protective effect^a^	References
*Megalocytivirus*								
ISKNV	Mandarin fish		Inactivated	ORF099	IP	China	Non-protective	[128]
	Mandarin fish			MCP, ORF054, ORF055, ORF101, ORF117, ORF125	IP	China	High	[94]
	Grouper			WCIV	IP	China	High	[129]
	Mandarin fish		Attenuated	ORF022L	IP	China	High	[130]
	Nile tilapia		Subunit	MCP	IP	Thailand	^b^	[100]
	Mandarin fish			Helicase ORF086	IP	China	High	[99]
	Mandarin fish			MCP	IMM	China	High	[102]
	Mandarin fish		DNA	MCP	IMM	China	High	[101]
	Chinese perch			MCP	IM	China	High	[97]
	Mandarin fish		Recombinant	MCP	IP	China	High	[106]
	Largemouth bass		Recombinant live-vector	MCP (Baculovirus)	IP or IMM	China	High	[104]
	Chinese perch			Helicase (*A. hydrophila*)	IP	China	High	[131]
RSIV	Rock bream		Inactivated	WCIV	IP	China	Moderate	[88]
	Red sea bream^b^ Striped jack^b^			WCIV WCIV	IP	Japan Japan	High	[85, 86] [87, 88]
	Rock bream		Live	Rearing Temperature	IM	Republic of Korea	Moderate	[132]
	Japanese flounder		DNA	MCP	IM	Japan	^b^	[133]
	Rock bream			Integrin	IP	Republic of Korea	^b^	[90]
	Red sea bream			MCP Transmembrane domain	IM	Japan	High	[91]
	Red sea bream		Recombinant live-vector	ORF18R, ORF351R, MCP (*E. coli*)	IP	Japan	High	[89]
	Red sea bream			ORF380R (*S. cerevisiae*)	O	Japan	^b^	[134]
TGIV	Grouper		Recombinant	P2 (*E. coli*)	IMM	China	High	[135]
RBIV	Rock bream		Inactivated	WCIV	IP	Republic of Korea	High	[106]
	Rock bream		DNA	Ankyrin	IM	Republic of Korea	Moderate	[109]
	Rock bream			MMP	IM	Republic of Korea	High	[108]
	Turbot			ORF75	IM	China	High	[136]
	Turbot			P86	IM	China	High	[137]
	Rock bream		Recombinant live-vector	MCP (*P. pastoris*)	O	Republic of Korea	High	[138]
OMIV	Marbled sleepy goby		Inactivated	WCIV	IP	China	High	[111]
SDDV	Asian seabass		Inactivated	WCIV	IP	The Netherlands	High	[139]
	Asian seabass		Recombinant Recombinant	MCP (*E. coli*) MCP (Baculovirus)	IP O	Thailand	^b^	[140]
*Lymphocystivirus*								
LCDV2	Japanese flounder		Inactivated	WCIV	IP	Republic of Korea	^b^	[141]
	Japanese flounder		DNA	MCP	IM, HD	China	High	[118]
	Japanese flounder		DNA	MCP	O	China	High	[113, 114]
LCDV3	Gilthead sea bream		DNA	MCP	IM	Spain	High	[67]
*Ranavirus*								
SGIV	Grouper		Inactivated	WCIV	IP	China	High	[119]
	Grouper		DNA	ORF19R	IM	China	High	[121]
LMBV	Largemouth bass		Subunit	MCP	IMM	China	High	[142]
	Largemouth bass		Recombinant live-vector	MCP (*P. pastoris*)	O	China	Moderate	[143]
	Largemouth bass			MCP (*S. cerevisiae*)	O	China	Moderate	[144]
	Largemouth bass			MCP (*B. subtilis*)	O	China	Moderate	[145]
	Largemouth bass		DNA	MCP	HD	China	Moderate	[123]

ISKNV: Infectious spleen and kidney necrosis virus; RSIV: Red sea bream iridovirus; TGIV: grouper iridovirus of Taiwan; RBIV: Rock bream iridovirus; SDDV: Scale drop disease virus; LCDV: Lymphocystis disease virus; SGIV: Singapore grouper iridovirus; LMBV: Largemouth bass ranavirus; OMIV: *Oxyeleotris marmoratus iridovirus*; IM: Intramuscular injection; IP: Intraperitoneal injection; IMM: Immersion; O: Oral; HD: Hypodermic; WCIV: Whole cell inactive vaccine.

^a^Protective effect: Ability of the vaccine to confer immunity against a specific infectious disease and is expressed as a percentage of protection.

^b^Protection must be demonstrated.

#### Megalocytivirus vaccines

Nakajima et al. found that a formalin-inactivated vaccine against RSIV (genotype II, clade 2) infection reduced mortality in red sea bream. This protective effect was due to the enhanced fish-specific immune response [85, 86]. In a subsequent study, Caipang et al. observed that using the same vaccine, virus, and fish host resulted in an increase in serum levels of neutralising antibodies and enhanced expression of MHC class I. This occurred when fish were vaccinated with either the intact formalin-inactivated vaccine or its protein derivatives [87]. However, only those fish vaccinated with the intact vaccine survived the viral challenge. This suggests that the fish’s survival was due to cell-mediated immunity, not serum-neutralizing antibodies. Subsequently, Kwon et al. prepared a formalin-inactivated RSIV (genotype II, clade 2) vaccine, and rock bream specimens were injected with different doses of the megalocytivirus vaccine formulations, with or without squalene or aluminum hydroxide as an adjuvant [88]. The results showed no differences in neutralising antibody titers. Vaccine efficacy and fish survival rates were dependent on vaccine dose and temperature.

Shimmoto et al*.* investigated the immunogenicity of subunit vaccines against RSIV (genotype II, clade 2) infection using three viral capsid proteins (18R, 351R, and MCP) [89]. Juveniles of red sea bream (*Pagrus major*) were intraperitoneally (IP) vaccinated with the recombinant formalin-killed *Escherichia coli* cells expressing these capsid proteins. They then underwent challenge infection with RSIV. Higher survival rates were observed in fish that received the 351R vaccine compared to the unvaccinated control group. A viral protein (351-R) was co-expressed with the bacterial glyceraldehyde 3-phosphate dehydrogenase as a fusion protein and showed improved protection against RSIV infection, leading to higher survival rates and increased levels of neutralising antibody levels.

Park et al. constructed a DNA vaccine by cloning the ORF 055L of RSIV into a plasmid containing a cytomegalovirus promoter. [90]. They investigated the ability of the pcDNA-055 DNA vaccine to induce neutralising antibodies against RSIV, determining the antibody efficacy using virus-inoculated BF-2 cell cultures. In another study, a DNA vaccine containing a plasmid encoding the MCP and an ORF of RSIV was developed and tested in red seabream by Caipang et al*.* [91]. MHC class I transcript expression increased in vaccinated fish. This pattern of expression was similar to that previously obtained by the same authors using a formalin-inactivated RSIV vaccine [87].

Fan et al. developed a formalin-inactivated vaccine against TRBIV (genotype III, clade 1) in turbots [92]. IM administration of the vaccine produced a high number of neutralising antibodies in the fish. The authors found that both subcutaneous and bath administration of the vaccine caused a drastic reduction in fish mortality. Zheng et al. developed a DNA vaccine against TRBIV [93]. Individual turbots were inoculated with a recombinant plasmid based on a eukaryotic plasmid (pVAX1) carrying a fragment of the *mcp* gene containing two antigenic epitopes of TRBIV. The vaccine induced both specific and non-specific cellular and humoral immune responses. In vaccinated fish, the activated up-regulating TFN, Mx, CXCR, and IFN expressions trigger an antiviral immune response in the host. In addition, after a TRBIV challenge, the vaccinated fish showed a higher survival rate, producing specific serum antibodies.

Dong et al. developed a formalin-killed cell-cultured vaccine against ISKNV (genotype I, clade 1) that protected more than 90% of vaccinated mandarin fish (*Synchiropus splendidus*) [94]. IgM-mediated immunity was the main response in vaccinated fish. In addition, six proteins were characterised as potent immunogens of ISKNV that specifically reacted with the sera antibodies. Zeng et al. reported the results of a gene-deleted live attenuated vaccine (ΔORF022L) in mandarin fish against ISKNV [95]. Vaccine protection showed a dose-dependent response, with 100% survival achieved at higher doses in vaccinated fish challenged with ISKNV. In addition, the vaccine induced anti-ISKNV-specific neutralising antibody responses, mainly IgM.

For ISKNV, Fu et al*.* cloned the MCP gene of this megalocytivirus into a prokaryotic expression vector pBV220 [96]. Juvenile mandarin fish were vaccinated via IP with recombinant MCP and an adjuvant, leading to high levels of specific antibodies and lymphocyte proliferation in vaccinated fish. However, the immune response was found to be dose-dependent. In another study by the same authors, the *mcp* gene of ISKNV was cloned into a eukaryotic expression vector pcDNA3.1 + (pcMCP) [97]. The immune response was induced by the IM injection of Chinese perch (*Siniperca chuatsi*) with pcMCP supplemented with the QCDC adjuvant. The expression levels of type I IFN system genes, including IRF-7, IRAK1, Mx, and Viperin, were up-regulated at 6 h, while a second peak in the expression levels of IRF-7 and Mx gene was obtained at 21 days post-vaccination. In addition, a remarkable increase in IgM levels was noted. The relative percentage survival (RPS) of Chinese perch vaccinated with pcMCP supplemented with adjuvant was 80% at 28 days post-vaccination. Later, Li et al*.* cloned the ORF093 gene of ISKNV, a predicted transmembrane protein, into a eukaryotic expression vector [pcDNA3.1. (+)] [98]. The efficacy of the vaccine was high compared to unvaccinated Chinese perch, with an RPS of ~ 51%.

Fu et al. [99] cloned the ORF086 gene, encoding an early protein helicase of ISKNV, into the prokaryotic pET32a (+) and the eukaryotic pcDNA3.1 (+) expression vectors. The mortality rate of vaccinated mandarins was reduced, with a survival rate of more than 63%. Transcriptional analysis of non-specific and specific immune-related genes revealed that IRF-7, IRAK1, Mx, Viperin, and IgM expression levels were strongly up-regulated in the vaccinated groups after immunisation. Throngnumchai et al. developed an ISKNV subunit vaccine by cloning the MCP gene of ISKNV into *E. coli* [100]. Nile tilapia specimens immunised with recombinant MCP showed significantly higher serum antibody titres than the control. In addition, the main immune-related genes activated in the spleen and the kidney in immunised fish corresponded to *mhcI*, *mhcII*, *il1β*, and *il4*.

Single-walled carbon nanotubes (SWCNTs) were used as a candidate for the ISKNV-DNA vaccine carrier to develop an immersion vaccine for juvenile mandarin fish [101]. The immune response obtained (immune-related gene expression, serum antibody production, enzyme activities, and C3 levels) was significantly enhanced in fish that had been vaccinated with SWCNTs-pcDNA-MCP compared to those vaccinated without the carrier. After 14 days of vaccination, the RPS reached 82.4% with SWCNTs-pcDNA-MCP, while only 54.2% was achieved in fish vaccinated with naked pcDNA-MCP. Zhao et al. developed another study based on the SWCNT-based subunit vaccine system (SWCNT-MCP) encoding the MCP gene of ISKNV [102]. A stronger and longer-lasting immune response (serum antibody production, enzyme activities, and immune-related gene expression) was induced in juvenile mandarin fish vaccinated by immersion compared to those vaccinated with MCP alone. Moreover, additional research has underscored the immune-potentiating impact of SWCNT employed in subunit vaccines against TGIV. This enhancement is demonstrated by increased survival rates due to higher antibody levels, improved activity of specific enzymes such as superoxide dismutase, alkaline phosphatase, and acid phosphatase, and the up-regulation of the immune-related gene expression [103].

Zhu et al. [104] used baculovirus technology to develop a live vector vaccine, BacMCP, containing the MCP coding sequence of ISKNV and driven by a CMV promoter. Immune-related genes (*IgM*, *tgfβ*, *il1β*, *il8*, *tnfα*) were overexpressed in BacMCP-vaccinated groups of largemouth bass (*Micropterus salmoides*). Vaccine efficacy depended on the route of inoculation and fish size, being 100% in small largemouth bass.

Shin et al. expressed a recombinant major capsid protein (rMCP) of RBIV in transgenic rice callus [105]. The rock bream specimens were immunised by feeding the rMCP in lyophilised rice callus powder. This vaccine induced intestinal mucosal immunity, as evidenced by higher serum IgM titers against rMCP in vaccinated fish and also by an increased survival rate after the iridovirus infection. Jung et al. evaluated the efficacy of squalene, aluminium hydroxide, and saponin as adjuvants of an inactivated vaccine to protect rock bream against RBIV infection [106]. Saponin induced an antiviral immune response (*il1ß*, *mx*, and *pkr* gene transcripts) and increased the survival rate of vaccinated fish after RBIV infection without producing side effects and with immunological memory. In addition, Ahn et al. engineered a plant-based vaccine expressing a recombinant MCP from RSIV combined with surface display on *Lactococcus lactis (L. lactis).* The plant-produced MCP, when displayed on *L. lactis*, was evaluated in mice models to assess its immunogenicity. The study demonstrated a strong immune response indicated by dose-dependent ELISA signal intensities [107].

In another study, Jung et al. [108] investigated the potential efficacy of a viral membrane protein (ORF008L) to protect rock bream from RBIV infection. Fish vaccinated with ORF008L-based DNA showed significant protection at four and eight weeks post-vaccination, significantly inducing the gene expression of *tlr3*, *MyD88*, *mx*, *isg15*, *pkr*, *mhcI*, *Fas*, *Fas* ligand, *caspase 9* and *3*. The same authors evaluated the ankyrin (ANK) repeat-containing proteins to induce protective immunity in RBIV-infected rock bream [109]. Fish were vaccinated with an ANK-based DNA vaccine and infected with RBIV by IP four to eight weeks after vaccination. At 7 days post-vaccination, the DNA vaccine induced an immune response characterised by the activation of genes related to TLR, IFN, and apoptosis pathways (*tlr3*, *tlr9*, *MyD88*, *mx*, *isg15*, *pkr*, *mhcI*, *perforin*, *Fas*, *Fas ligand*, and *caspase 8*,* 9*, and *3*). The levels of inflammatory cytokines gene expression (*il1ß*, *il*8, and *tnf*α) did not increase in the vaccinated fish. High protection was initially achieved in the vaccinated group at four weeks post-vaccination, but it decreased over time. The authors found that 100% of the vaccinated fish survived reinfection with a higher concentration of RBIV.

Mahardika and Mastuti produced a crude subunit vaccine using the MCP of GSDIV (genotype II, clade 2), which was inserted into an expression system vector and cloned into *E. coli* [110]. *E. coli* that expressed the MCP protein was inactivated with formalin, and this crude subunit vaccine was IM injected into humpback grouper (*Cromileptes altivelis*) specimens. The vaccination improved virus protection and reduced fish mortality.

Guo et al. investigated the protective effect of a bivalent inactivated vaccine developed against Oxyeleotris marmoratus iridovirus (OMIV, genotype II) [111]. A transcriptomic profiling study was conducted using RNA sequencing of spleen tissues at various time points after vaccination and OMIV infection. After immunisation, the immune response led to increased production of pro-inflammatory cytokines (such as IL12). Furthermore, it triggered both cell-mediated and humoral immune responses by activating CD8 + cells, TCR, MHC I, MHC II, IgM, and CD4 + cells. The authors noted a significant increase in survival rate, reaching 100% after a lethal dose. This led to a secondary immune response characterised by the activation of cellular and humoral immunity seven days after the OMIV challenge.

#### Lymphocystivirus vaccines

Zheng et al. investigated the expression of a DNA vaccine (pEGFP-N2-LCDV 0.6 kb) against LCDV in Japanese flounder [112]. This vaccine prevented the development of tumour growth in vaccinated fish. Later, Tian et al*.* formulated a DNA vaccine against LCDV contained in PLGA microcapsules to prevent DNA denaturation by nucleases in the gastrointestinal tract of Japanese flounder [113]. The microencapsulated vaccine significantly increased the serum of specific antibodies against LCDV for up to 24 weeks. The same authors prepared nanoparticles of PLGA to encapsulate the developed vaccine against LCDV for the oral immunisation of Japanese flounders [114]. The nanoparticle vaccine stimulated the immune response of the fish by increasing antibody, superoxide dismutase, and lysozyme levels, and also by activating phagocytosis.

Zheng et al. [115] developed a DNA vaccine carrying the LCDV *mcp* gene (pEGFP-N2-LCDV 0.6 kb) against LCDV-C. Vaccinating Japanese flounder via IM injection activated genes involved in the inflammatory process and triggered specific cellular and humoral immune responses. Later, the same authors tested this DNA vaccine in Japanese flounder specimens using two routes: IM and subcutaneous injection [116]. The results obtained suggest that the humoral and cell-mediated immune responses depend on the route of vaccination used.

A new DNA vaccine against LCDV-Sa was developed by Leiva-Rebollo et al. The vaccine was constructed by cloning the *mcp* gene into a plasmid and applied to gilthead seabream specimens via the IM route [117]. This vaccine induces an immune response characterised by the overexpression of genes involved in the inflammatory process and induces a humoral immune response characterised by the production of specific neutralising antibodies. In another study, the same authors [118] showed that the DNA vaccine induced an immune response in gilthead seabream, characterised by an increase in deregulated genes in the hematopoietic organs of vaccinated fish. Using the OpenArray^®^ platform, fish vaccination significantly reduced virus replication in the vaccinated fish and resulted in the expression of immune genes related to virus recognition (*tlr9*), humoral and cellular response (*rag1* and *cd48*), inflammation (*csf1r, elam, il1β*, and *il6*), antiviral response (*isg15, mx1, mx2, mx3*), cell-mediated cytotoxicity (*nccrp1*), and apoptosis (*prf1*). The exclusive modulation of the immune response induced by the vaccination seems to control the progression of the infection in the experimentally challenged gilthead seabream.

#### Ranavirus vaccines

Ranavirus vaccines have so far been scarcely developed and studied. Orange-spotted groupers vaccinated with two inactivated ranavirus SGIV formulations showed high effectiveness, with over 90% survival rates among immunised fish [119]. Both vaccines induced a non-specific antiviral immune response characterised by the expression of pro-inflammatory cytokines and IFN-I-stimulated genes (*mx1* and *isg15*). One month after vaccination, the vaccine induces specific humoral and cellular immune responses through the activation of MHC class I and cytokine (*il8*) genes and by the production of specific serum antibodies. Ma et al. developed a water-in-oil formulation of a formalin-killed bivalent vaccine (GrouperVAC-Irido-R-Vh) against the ranavirus GIV [120]. The vaccine, inoculated by IP injection into juvenile groupers, showed a protective role against the ranavirus infection. Yu et al. [121] developed an SGIV ORF19R (SGIV-19R) encoding viral membrane protein constructed in pcDNA3.1-HA and used it to evaluate the immunoprotective effects in groupers. Transcript levels of *tnfα*, *il1β*, *mx1*, and *IgM* genes were significantly up-regulated in the spleen, liver, and kidney of vaccinated groupers. However, SGIV challenge experiments showed that the relative percentage of survival induced by the vaccine ranged from 49.9% to 75%. Another study was conducted to develop an effective vaccine against SGIV using spores of *Bacillus subtilis* WB600 as a vehicle for the VP19 protein displayed on the surface administrated orally to groupers. This vaccine generated not activated innate immunity but also induced cellular immunity and antiviral activity, thus increasing the survival rate [122].

Yi et al. [123] constructed a DNA vaccine by inserting the cloned largemouth bass virus (LMBV)-*mcp* gene into the pCDNA3.1(+)-flag plasmid. The DNA vaccinated group of the largemouth bass group showed significantly up-regulated expression of *il1β*, *il8*, *tnfα,* and *mx* genes in the spleen, head kidney, and liver. All fish immunised with the DNA vaccine produced a high titre of LMBV-specific neutralising antibodies during the immunisation period.

## Conclusions

The research outlined in this review demonstrates unequivocally that immunity against iridoviruses is multifaceted and intricate in nature and is probably influenced by species and developmental stage specificity. Furthermore, these investigations reveal significant gaps in our comprehension of the immune responses these pathogens elicit, as well as potential deficiencies in the host capacity to generate robust defenses capable of controlling and eradicating such infections. The family *Iridoviridae’s* numerous, highly effective mechanisms for evading host immune components are of particular concern. This promotes viral persistence, facilitates dissemination, and broadens the host range. Based on the existing data on the immune response triggered by iridovirus infection, most infections lead to a systematic decrease in inflammatory responses and suppression of the adaptive immune system. Considering these observations, it seems that the virus may be using certain mechanisms to avoid detection by the host immune system. Understanding these immune evasion pathways may aid in developing new vaccination strategies against various viruses from the *Iridoviridae* family.

Currently, the availability of fish virus vaccines is very limited, and the licensed vaccines cannot fully meet the needs of the aquaculture industry. Therefore, more efforts are required to meet aquaculture needs by intensifying research into the development of highly effective aquatic vaccines.

In the case of iridovirus vaccines, future research will focus on understanding the mechanism of mucosal immunity and its relationship with systemic immunity. In addition, further studies are needed on antigen processing for vaccine production, effective doses, vaccine-coating materials and carriers, and adjuvants. Other important aspects to be considered include the duration of immunisation, the number of boosters, and the physiology and developmental stages of the fish. It is also necessary to improve the evaluation system of fish vaccines by detecting changes in gene and protein levels, as well as antibody and cellular responses in vaccinated fish.

The selection of appropriate viral antigens is critical for the optimal vaccine design. The rapid development of omics such as genome, functional genome, proteome, and metabolome, along with genome editing technologies, may offer crucial insights into the genomes of fish iridoviruses, their infection process, and the identification of genetic targets for highly effective vaccines. It is clear that nucleic acid-based vaccines, such as DNA vaccines, live vector vaccines, and mRNA vaccines will play a significant role in preventing viral infectious diseases in aquaculture.

### Supplementary Information

**Additional file 1. Marine and freshwater (including ornamental) fish susceptible to ***Iridoviridae*** infections.**

## Data Availability

Not applicable.
