# Supplementary material for: Fish Iridoviridae: infection, vaccination and immune response
Source: Vet Res. 2024 Jul 15;55:88. doi: 10.1186/s13567-024-01347-1 (PMC11247874; doi:10.1186/s13567-024-01347-1)
Supplement: Supplementary file 1 — Additional file 1. Marine and freshwater (including ornamental) fish susceptible to Iridoviridae infections. [file 13567_2024_1347_MOESM1_ESM.docx]

**Additional file 1. Marine and freshwater (including ornamental) fish susceptible to *Iridoviridae* infections**

| Fish Order/family | Common name | | Scientific name | | Virus infection |  |
| --- | --- | --- | --- | --- | --- | --- |
| **O. Acipenseriformes** |  | |  | |  |  |
| *Acipenseridae* | Russian sturgeon | | *Acipenser gueldenstaedtii* | | *Ranavirus* (FV3) |  |
|  | White sturgeon | | *A. transmontanus* | | *Ranavirus* (WSIV) |  |
|  | Amur sturgeon | | *A. schrenckii* | | *Ranavirus* (FV3) |  |
|  | Pallid sturgeon | | *Scaphirhynchus albus* | | *Ranavirus* (FV3) |  |
| **O. Anabantiformes** |  | |  | |  |  |
| *Osphronemidae* | Siamese fighting fish | | *Betta splendens* | | *Megalocytivirus* |  |
|  | Dwarf gourami | | *Colisa lalia* | | *Megalocytivirus* (DGIV) |  |
|  | Thick-lipped gourami | | *C. labiosa* | | *Megalocytivirus* (ISKNV) |  |
|  | Banded gourami | | *C. fasciata* | | *Megalocytivirus* |  |
|  | Three-spot gourami | | *Trichogaster trichopterus* | | *Megalocytivirus* |  |
|  | Pearl gourami | | *T. leerii* | | *Megalocytivirus* (ISKNV) |  |
|  | Moonlight gourami | | *T. microlepsis* | | *Megalocytivirus* |  |
|  | Roundtail Paradise fish | | *Macropodus opercularis* | | *Megalocytivirus* (RSIV/ISKNV) |  |
| **O. Anguilliformes** |  | |  | |  |  |
| *Anguillidae* | Short-finned eel | | *Anguilla australis* | | *Ranavirus* (SERV) |  |
| **O. Characiformes** |  | |  | |  |  |
| *Characidae* | Neon tetra | | *Paracheirodon innesi* | | *Megalocytivirus* |  |
| **O. Cichliformes** |  | |  | |  |  |
| *Cichlidae* | Oscar | | *Astronotus ocellatus* | | *Megalocytivirus* (ISKNV/TRBIV) |  |
|  | Orange chromide | | *Etroplus maculates* | | *Megalocytivirus* |  |
|  | Banded cichlid | | *Heros severus* | | *Megalocytivirus* |  |
|  | Blue ram | | *Mikrogeophagus ramirezi* | | *Megalocytivirus* (ISKNV) |  |
|  | Freshwater angelfish | | *Pterophyllum scalare* | | *Megalocytivirus* (ISKNV) |  |
| **O. Clupeiformes** |  | |  | |  |  |
| *Clupeidae* | Paciﬁc herring | | *Clupea pallasii* | | *Lymphocystivirus* |  |
| **O. Cypriniformes**  *Cyprinidae* | Zebrafish | | *Danio rerio* | | *Megalocytivirus* (ISKNV) |  |
|  | Common carp | | *Cyprinus carpio* | | *Ranavirus* (CCIV) |  |
|  | Bluehead chub | | *Nocomis leptocephalus* | | *Ranavirus* |  |
| **O. Cyprinodontiformes**  *Aplocheilidae* | African lampeye | | *Aplocheilichthys normani* | | *Megalocytivirus* (ISKNV) |  |
| *Nothobranchiidae* | Steel blue killifish | | *Fundulopanchax* *gardneri* | | *Megalocytivirus* (ISKNV) |  |
| *Poeciliidae* | Green swordtail | | *Xiphophorus hellerii* | | *Megalocytivirus* (ISKNV) |  |
|  | Common platy | | *X. maculatus* | | *Megalocytivirus* (ISKNV) |  |
|  | Variable platyfish | | *X. variatus* | | *Megalocytivirus* (ISKNV) |  |
|  | Sailfin molly | | *Poecilia latipinna* | | *Megalocytivirus* (ISKNV) |  |
|  | Guppy | | *P. reticulata* | | *Megalocytivirus* (ISKNV)  *Ranavirus* (GV6) |  |
|  | Common molly | | *P. sphenops* | | *Megalocytivirus* (ISKNV) |  |
|  | Norman’s lampeye | | *Aplocheilichthys normani* | | *Megalocytivirus* (ISKNV) |  |
|  | Mosquitofish | | *Gambusia affinis* | | *Ranavirus* (EHNV/ FV3) |  |
| **O. Esociformes** |  | |  | |  |  |
| *Esocidae* | Chain pickerel | | *Esox niger* | | *Ranavirus* (LMBV) |  |
|  | Muskellunge | | *E. masquinongy* | | *Ranavirus* |  |
|  | Pike | | *E. lucius* | | *Ranavirus* (EHNV/ FV3) |  |
| **O. Gadiformes** |  | |  | |  |  |
| *Gadidae* | Cod | | *Gadus morhua* | | *Ranavirus* (CoIV) |  |
| **O. Galaxiiformes** |  | |  | |  |  |
| *Galaxiidae* | Mountain galaxias | | *Galaxias olidus* | | *Ranavirus* (EHNV) |  |
| **O. Gobiiformes** |  | |  | |  |  |
| *Gobiidae* | Marbled sleepy goby | | *Oxyeleotris marmoratus* | | *Megalocytivirus* (ISKNV) |  |
| **O. Labriformes** | |  |  |  | | |
| *Labridae* | | Bluestreak cleaner wrasse | *Labroides dimidiatus* | *Ranavirus* (DFV) | | |
| **O. Mugiliformes**  *Mugilidae* | | Gray mullet | *Mugil cephalus* | *Megalocytivirus* (RSIV | | |
| **O. Perciformes** | |  |  |  | | |
| *Acanthuridae* | | Doctorfish | *Acanthurus chirurgus* | *Ranavirus* | | |
| *Ambassidae* | | Painted glass fish | *Parambassis baculis* | *Lymphocystivirus* (LCDV-PaGl) | | |
| *Apogonidae* | | Banggai cardinalfish | *Pterapogon kauderni* | *Megalocytivirus* (ISKNV) | | |
| *Carangidae* | | Japanese amberjack | *Seriola quinqueradiata* | *Megalocytivirus* (RSIV)  *Lymphocystivirus* | | |
|  | | Yellowtail amberjack | *S. lalandi* | *Megalocytivirus* (RSIV) | | |
|  | | Greater amberjack | *S. dumerili* | *Megalocytivirus* (RSIV) | | |
|  | | Goldstriped amberjack | *S. aureovittata* | *Megalocytivirus* (RSIV | | |
|  | | Striped jack | *Pseudocaranx dentex* | *Megalocytivirus* (RSIV) | | |
|  | | Snubnose pompano | *Trachinotus blochii* | *Megalocytivirus* (RSIV) | | |
|  | | *Florida pompano* | *T. carolinus* | *Megalocytivirus* (RSIV) | | |
|  | | Japanese jack mackerel | *Trachurus japonicus* | *Megalocytivirus* (RSIV) | | |
|  | | Common jack | *Caranx hippos* | *Megalocytivirus* | | |
| *Centrarchidae* | | Redbreast sunfish | *Lepomis auritus* | *Ranavirus* | | |
|  | | Bluegill | *L. macrochirus* | *Ranavirus* (LMBV) | | |
|  | | Longear sunfish | *L. megalotis* | *Ranavirus* | | |
|  | | Black crappie | *Pomoxis nigromaculatus* | *Ranavirus* (LMBV) | | |
|  | | Rock bass | *Ambloplites rupestris* | *Ranavirus* | | |
| *Centropomidae* | | Barramundi | *Lates calcarifer* | *Megalocytivirus* (RSIV/ISKNV/TRBIV/SDDV)  *Lymphocystivirus* (LCDV-1)  *Ranavirus* (BIV) | | |
|  | | Mandarinfish | *Siniperca chuatsi* | *Megalocytivirus* (RSIV/ISKNV) | | |
|  | | Largemouth bass | *Micropterus salmoides* | *Megalocytivirus* (ISKNV)  *Lymphocystivirus* (Leetown NFH)  *Ranavirus* (LMBV) | | |
|  | | Suwannee bass | *M. notius* | *Ranavirus* (LMBV) | | |
|  | | Spotted bass | *M. punctulatus* | *Ranavirus* (LMBV) | | |
| *Cichlidae* | | Red tiger oscar | *Astronotus ocellatus* | *Megalocytivirus* (ISKNV/TRBIV) | | |
|  | | Freshwater angelfish | *Pterophyllum scalare* | *Megalocytivirus* (ISKNV/TRBIV) | | |
|  | | Nile tilapia | *Oreochromis niloticus* | *Megalocytivirus* (ISKNV)  *Ranavirus* (BIV) | | |
| *Eleotridae* | | Marble goby | *Oxyeleotris marmoratus* | *Megalocytivirus* (ISKNV)  *Ranavirus* (OMRV) | | |
| *Ephippidae* | | Batfish | *Platax orbicularis* | *Megalocytivirus*  (ISKNV) | | |
| *Haemulidae* | | Threeline grunt | *Parapristipoma* *trilineatum* | *Megalocytivirus* (RSIV) | | |
|  | | Threeband sweetlips | *Plectorhynchus* *cinctus* | *Megalocytivirus* (RSIV) | | |
| *Kyphosidae* | | Largescale blackfish | *Girella punctata* | *Megalocytivirus* (RSIV) | | |
| *Leiognathidae* | | Common ponyfish | *Leiognathus equulus* | *Megalocytivirus* (RSIV) | | |
| *Lethrinidae* | | Chinese emperor | *Lethrinus haematopterus* | *Megalocytivirus* (RSIV) | | |
| *Lutjanidae* | | Crimson snapper | *Lutjanus erythropterus* | *Ranavirus* | | |
| *Moronidae* | | Japanese sea perch/sea bass | *Lateolabrax japonicus* | *Megalocytivirus* (RSIV)  *Lymphocystivirus* (LCDV-SB) | | |
|  | | Striped bass | *Morone saxatilis* | *Megalocytivirus* (RSIV)  *Ranavirus* (SCRV) | | |
|  | | White bass | *M. chrysops* | *Ranavirus* | | |
|  | | Yellow bass | *M. mississippiensis* | *Ranavirus* | | |
| *Oplegnathidae* | | Rock bream | *Oplegnathus fasciatus* | *Megalocytivirus* (RSIV/TRBIV)  *Ranavirus* | | |
| *Oplegnathidae* | Spotted knifejaw | | *O. punctatus* | | *Megalocytivirus* (RSIV) |  |
| *Osphronemidae* | Dwarf gourami | | *Colisa lalia* | | *Megalocytivirus* (ISKNV) |  |
|  | Pearl gourami | | *Trichogaster leeri* | | *Megalocytivirus* (ISKNV)  *Lymphocystivirus* (LCDV-PeGr) |  |
|  | Silver gourami | | *T. microlepis* | | *Megalocytivirus* (ISKNV) |  |
|  | Snakeskin gourami | | *T. pectoralis* | | *Lymphocystivirus** |  |
|  | Three-spot gourami | | *T. trichopterus* | | *Lymphocystivirus** |  |
| *Percichthyidae* | Murray cod | | *Macullochella peelii* | | *Megalocytivirus* (ISKNV)  *Ranavirus* (EHNV) |  |
|  | Macquarie perch | | *Macquaria australasica* | | *Ranavirus* (EHNV) |  |
|  | Golden perch | | *M. ambigua* | | *Ranavirus* (EHNV) |  |
|  | Australian bass | | *M. novemaculeata* | | *Ranavirus* (EHNV) |  |
| *Percidae* | Yellow perch | | *Perca flavescens* | | *Lymphocystivirus* (YP1) |  |
|  | Redfin perch | | *P. fluviatilis* | | *Ranavirus* (EHNV) |  |
|  | Pike-perch | | *Sander lucioperca* | | *Ranavirus* (EHNV/FV3) |  |
| *Pomacentridae* | Clown anemonefish | | *Amphiprion ocellaris* | | *Lymphocystivirus* (LCDV-1) |  |
| *Rachycentridae* | Cobia | | *Rachycentron canadum* | | *Megalocytivirus* (RSIV)  *Lymphocystivirus* (LCDV-RC |  |
| *Sciaenidae* | Croceine croaker | | *Larimichthys crocea* | | *Megalocytivirus* (RSIV) |  |
|  | Red drum | | *Sciaenops ocellatus* | | *Megalocytivirus* (ISKNV)  *Lymphocystivirus** |  |
|  | Atlantic croaker | | *Micropogon undulatus* | | *Lymphocystivirus** |  |
|  | Whitemouth croaker | | *Micropogonias furnieri* | | *Lymphocystivirus* (LCDV-4/LCDV-WC) |  |
|  | Freshwater drum | | *Aplodinotus grunniens* | | *Ranavirus* |  |
| *Scombridae* | Northern bluefin tuna | | *Thunnus thynnus* | | *Megalocytivirus* (RSIV) |  |
|  | Japanese mackerel | | *Scomberomorus niphonius* | | *Megalocytivirus* (RSIV) |  |
|  | Chub mackerel | | *Scomber japonicus* | | *Megalocytivirus* (RSIV) |  |
|  |  | |  | |  |  |
| *Serranidae* | Tiger grouper | | *Epinephelus fuscoguttatus* | | *Megalocytivirus* (RSIV)  *Lymphocystivirus* (GLCDV) |  |
|  | Banded grouper | | *E. awoara* | | *Megalocytivirus* (RSIV)  *Ranavirus* (GIV/SGIV) |  |
|  | Red spotted grouper | | *E. akaara* | | *Megalocytivirus* (RSIV) |  |
|  | Sevenbanded grouper | | *E. septemfasciatus* | | *Megalocytivirus* (RSIV) |  |
|  | Brown-spotted grouper | | *E. malabaricus* | | *Megalocytivirus* (RSIV)  *Ranavirus* (SGIV) |  |
|  | Giant grouper | | *E. lanceolatus* | | *Megalocytivirus*  (RSIV/ISKNV)  *Lymphocystivirus* (GLCDV)  *Ranavirus* (GIV-R) |  |
|  | Greasy grouper | | *E. tauvina* | | *Megalocytivirus*  (RSIV)  *Lymphocystivirus** |  |
|  | Kelp grouper | | *E. moara* | | *Megalocytivirus* (RSIV) |  |
|  | Orange-spotted grouper | | *E. coioides* | | *Megalocytivirus* (RSIV/ISKNV)  *Ranavirus* (SGIV) |  |
|  | Longtooth grouper | | *E. bruneus* | | *Megalocytivirus*  (RSIV) |  |
|  | Humpback grouper | | *Cromileptes altivelis* | | *Megalocytivirus* (RSIV/ISKNV) |  |
| *Sparidae* | Red sea bream | | *Pagrus major* | | *Megalocytivirus* (RSIV)  *Ranavirus*  *Lymphocystivirus** |  |
|  | Black sea bream | | *Acanthopagrus schlegeli* | | *Megalocytivirus* (RSIV) |  |
|  | Yellowfin sea bream | | *A. latus* | | *Megalocytivirus* (RSIV) |  |
|  | Crimson sea bream | | *Evynnis japonica* | | *Megalocytivirus*  (RSIV) |  |
|  | Silver sea bream | | *Rhabdosargus sarba* | | *Megalocytivirus* (RSIV/ISKNV) |  |
|  | Gilthead sea bream | | *Sparus aurata* | | *Lymphocystivirus* (LCDV-3/LCDV-Sa) |  |
| *Sparidae* | Blackspot sea bream | | *Pagellus bogaraveo* | | *Lymphocystivirus** |  |
| *Terapontidae* | Silver perch | | *Bidyanus bidyanus* | | *Ranavirus* (EHNV) |  |
|  |  | |  | |  |  |
| **O. Pleuronectiformes**  *Pleuronectidae* | Spotted halibut | | *Verasper variegatus* | | *Megalocytivirus* (TRBIV-like) |  |
|  | European plaice | | *Pleuronectes platessa* | | *Lymphocystivirus* (LCDV-1) |  |
|  | Common dab | | *Limanda limanda* | | *Lymphocystivirus* (LCDV-2) |  |
| *Scophthalmidae* | Turbot | | *Scophthalmus maximus* | | *Megalocytivirus* (TRBIV)  *Ranavirus* |  |
| *Paralichthyidae* | Japanese/ olive flounder | | *Paralichthys olivaceus* | | *Megalocytivirus* (RSIV)  *Lymphocystivirus*  **(**LCDV-C) |  |
|  | European flounder | | *P. flesus* | | *Lymphocystivirus* (LCDV-1) |  |
| *Soleidae* | Senegalese sole | | *Solea senegalensis* | | *Lymphocystivirus* (LCDV-SSE) |  |
| **O. Salmoniformes**  *Salmonidae* | Rainbow trout | | *Oncorhynchus mykiss* | | *Ranavirus* (EHNV) |  |
|  | Chinook salmon | | *O. tshawytscha* | | *Ranavirus* (SCRV) |  |
| **O. Scorpaeniformes**  *Scorpaenidae* | Rockfish | | *Sebastes schlegelii* | | *Megalocytivirus*  *Lymphocystivirus* (LCDV-RF) |  |
| *Triglidae* | Grey gurnard | | *Eutrigla gurnardus* | | *Lymphocystivirus* (LCDV-1) |  |
| *Gasterosteidae* | Three-spined stickleback | | *Gasterosteus aculeatus* | | *Ranavirus* (FV3) |  |
| *Cyclopteridae* | Lumpfish | | *Cyclopterus lumpus* | | *Ranavirus* (LfRV) |  |
| **O. Siluriformes** |  | |  | |  |  |
| *Ictaluridae* | Black bullhead | | *Ameiurus melas* | | *Ranavirus* (ECV/FV3) |  |
|  | Brown bullhead | | *A. nebulosus* | | *Ranavirus* (ESV) |  |
| *Siluridae* | European catfish/sheatfish | | *Silurus glanis* | | *Ranavirus* (ESV) |  |
| **O.Tetraodontiformes**  *Tetradontidae* | Tiger puffer | | *Takifugu rubripes* | | *Megalocytivirus* (RSIV-like) |  |

* Virus names and abbreviations are not official designations.
